# Current Treatment Practices and Prognostic Factors in Early-Stage Ovarian Cancer—An Analysis of the NOGGO/JAGO

**DOI:** 10.3390/cancers15072038

**Published:** 2023-03-29

**Authors:** Sabine Heublein, Joanna Baum, Anna Jaeger, Donata Grimm-Glang, Julia Olthoff, Elena Ioana Braicu, Osama Azzam Nieto, Kathrin Hassdenteufel, Barbara Schmalfeldt, Lars Hanker, Markus Wallwiener, Andreas Schneeweiss, Jalid Sehouli, Klaus Pietzner

**Affiliations:** 1 Department of Gynecology and Obstetrics, University of Heidelberg, 69120 Heidelberg, Germany; 2 National Center for Tumor Diseases, University Hospital and German Cancer Research Center Heidelberg, 69120 Heidelberg, Germany; 3 Young Academy of Gynecologic Oncology (JAGO), 13359 Berlin, Germany; 4 Department of Gynecology, Center for Oncological Surgery, Campus Virchow Klinikum, Charite-Universitätsmedizin Berlin, Corporate Member of Freie Universität Berlin, Humboldt-Universität zu Berlin, Berlin Institute of Health, 13353 Berlin, Germany; 5 Department of Gynecology and Gynecologic Oncology, University Medical Center Hamburg-Eppendorf, 20246 Hamburg, Germany; 6 Department of Obstetrics and Gynecology, University Medical Center Luebeck, 23562 Luebeck, Germany; 7 Department of Obstetrics and Gynecology, University Hospital Brandenburg an der Havel, 14770 Brandenburg an der Havel, Germany

**Keywords:** early-stage ovarian cancer, surgery, BRCA, adjuvant treatment

## Abstract

**Simple Summary:**

Patients diagnosed with early-stage ovarian cancer (OC) are treated by surgery and, if appropriate, by adjuvant chemotherapy. However, in practice, there is disagreement about both the extent of surgical staging and the indication of adjuvant chemotherapy. Therefore, we sought to structurally assess clinically relevant parameters of treatment practice and aimed to identify individual patient factors that may influence decisions regarding the extent of treatment. This study confirmed that a relevant proportion (34.2%) of early-stage OC patients were not operated on in compliance with the current standard. This was independent of patient age and hospital healthcare level. The reasons for omitting complete surgical staging were balanced. Only a subset of cases underwent *BRCA1/2* testing. Though mono-chemotherapy is recommended for early-stage OC, almost every second patient receives combination treatment. Our study underlines the high need for further prospective registries and educational programs to increase awareness for adequate clinical management of patients with early-stage OC.

**Abstract:**

Background: Surgery is the backbone of early-stage ovarian cancer (OC) management. However, in practice, there is disagreement about the extent of surgical staging and whether additional adjuvant treatment should be provided. As omitting relevant diagnostic or therapeutic procedures might lead to undertreatment, we aimed to structurally investigate treatment practice in addition to prognostic factors in a multicentre series of patients (pts) diagnosed with early-stage OC. Patients: Within this retrospective, multicentre study, medical records of 379 pts who had undergone surgery for suspected early-stage OC between January 2014 and March 2020 were analysed. Results: Of the 379 patients, 292 had pT stage 1a-2a and had complete data on the extent of surgical staging. At least one surgical step was omitted in 100 pts (34.2%). Complete surgical staging (*n* = 192; (65.8%)) was more often performed in high-grade serous OC and was independent of the healthcare level of the hospital where the initial diagnosis was made. Missing to take peritoneal biopsies was associated with reduced relapse-free-survival in incompletely staged, pT1 cases (*p* = 0.03). About every second patient (46.7%) with a final stage lower than FIGO IIB and treated with adjuvant chemotherapy received combination chemotherapy. *BRCA1* and *BRCA2* testing was only performed in a subset of pts, and mutations were detected in 18% (14/79) and 9% (7/85) pts, respectively. Conclusions: This study helps to increase our understanding of early-stage OC treatment and prognosis. In addition to treating patients in compliance with current guidelines, the need for *BRCA* testing should also be considered for early-stage OC.

## 1. Introduction

Approximately one-third of ovarian cancer (OC) patients are diagnosed at a time when the cancer is still limited to the pelvis [i.e., Federation of Gynaecology and Obstetrics (FIGO) IA to IIB] [1]. Although the overall prognosis for these patients is markedly better than those with more advanced disease, about 25% of them will experience disease relapse, and approximately 20% will even die due to OC [2,3]. Among all guidelines, it is widely accepted that surgery is the preferred treatment for early-stage OC. In Germany, optimal surgical staging has been defined by the national guideline, which is released by the German Society of Gynaecology and Obstetrics every 4 years [4]. According to the current guideline, surgical staging in early-stage OC should comprise laparotomy, bilateral adnexectomy, hysterectomy, omentectomy, pelvic and para-aortic lymphadenectomy, peritoneal sampling, cytology specimen retrieval, and appendectomy in the case of mucinous histology. As the guideline does not consider systematic lymphadenectomy to be obligatory for well-differentiated (Grade 1) mucinous disease, our definition of complete staging did not require systematic lymphadenectomy for them.

Complete surgical staging followed by stage-adapted adjuvant treatment significantly improves patients’ 4-year survival [5]. Omitting surgical steps is critical for two reasons. First, if cancer staging is suboptimal, its spread to a certain organ might remain undetected if the organ is not resected as recommended. This is especially true regarding microscopic spread. Therefore, underestimating cancer spread might lead to falsely classifying the patient as being in an early FIGO stage and prevent the patient from receiving adequate adjuvant chemotherapy. Second, through suboptimal staging, the surgeon accepts the risk of leaving tumour residuals in the body. Although the importance of optimal surgical staging seems reasonable and surgical steps are clearly defined by the guideline [4], a relevant number of early-stage OC patients are not treated in accordance with the official recommendations. This seems to be an international dilemma, as several reports from different countries claim that treatment standards are insufficiently applied in the case of early-stage OC [5,6]. Only recently, a nationwide survey by the QS Ovar revealed that only about 70% of patients diagnosed with early-stage OC were operated on in compliance with the national guideline [4,5]. Although this rate has improved over the last decade, approximately every third of patients diagnosed with apparent early-stage OC has not been treated according to the established standards [7].

Inspired by the results of the nationwide, descriptive analysis by the QS Ovar, we sought to structurally assess clinically relevant parameters of treatment practice in early-stage OC. Moreover, we aimed to identify individual patient factors (e.g., conservation of fertility and comorbidities) that may influence decisions regarding the extent of treatment. These analyses are fundamental to improving our understanding of treatment for early-stage OC. 

## 2. Materials and Methods

### 2.1. Characteristics of the Study Sample

This multicentre registry study retrospectively analysed medical records of patients diagnosed with early-stage OC. Centres participating in this study included the Department of Gynaecology and Gynaecologic Oncology, Charité University Hospital Berlin, the Department of Obstetrics and Gynaecology, University of Heidelberg, the Department of Gynaecology and Gynaecologic Oncology, University Medical Centre Hamburg—Eppendorf Hamburg, the Department of Obstetrics and Gynaecology, University Medical Centre Luebeck, and the Department of Obstetrics and Gynaecology, University Hospital Brandenburg an der Havel. This study was performed in collaboration with the JAGO (‘Young Academy of Gynaecologic Oncologists’) of the NOGGO (‘North-Eastern German Society of Gynaecological Oncology’) study group. 

All patients with apparent early-stage OC in whom surgical cancer resection was performed between January 2014 and March 2020 were included in this study. We decided to use pT rather than FIGO stage to pre-select patients with presumable early-stage disease in the first place. Selection by final FIGO stage (e.g., only analysing those patients with FIGO I and II) would have biased our results since those patients with microscopic lymphonodular or peritoneal spread are detected only after complete surgical staging (i.e., by systemic lymphadenectomy or peritoneal sampling) and hence finally up-staged as FIGO III would be missed. As a second selection step, clinical files, including surgery reports, were carefully analysed. Only cases with pT1a to pT2b without macroscopic peritoneal spread or in terms of pT2b without macroscopic peritoneal spread outside the pelvis were defined as apparently early-stage and analysed in this study.

**Figure 1:**
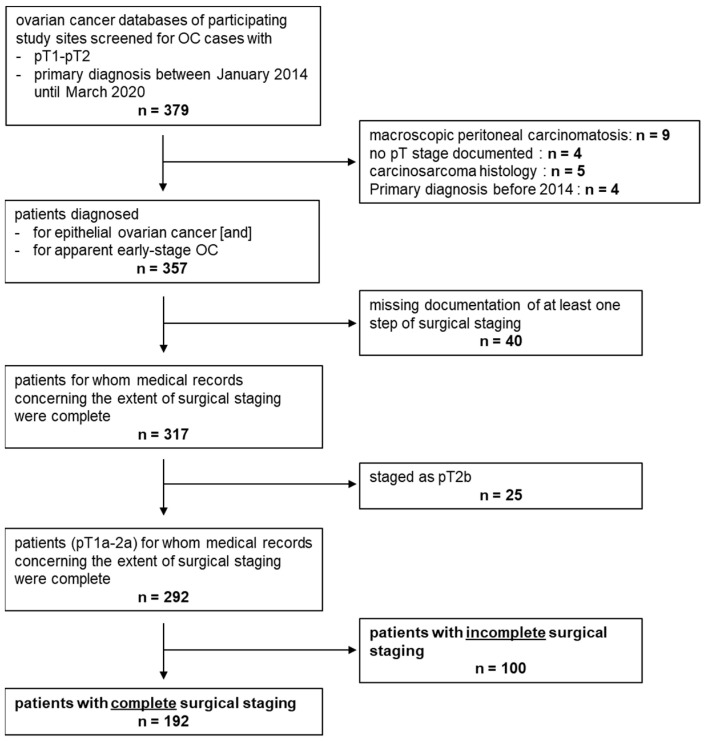
Consort Diagram. Data sets from 379 ovarian cancer patients with pT1-2 stage were collected. Since 22 cases finally did not fulfil inclusion criteria or had macroscopically visible peritoneal carcinomatosis outside the pelvis, they had to be excluded (Figure 1). In total, 357 patients with apparent early-stage OC were analysed. For analysing details on surgery, those patients with full medical records on surgical staging (*n* = 317) and—as long as not stated otherwise—apparent pT stage pT1-pT2a (*n* = 292) were selected (Figure 1).

Stage pT2b is not covered by the definition of early-stage OC as defined by the German guideline in its current version [4]. pT2b tumours range between clearly early (pT1a–pT2a) and obviously advanced staged (pT3, pT4). As most clinical trials focus on advanced staged OC and maintenance treatment is only approved for these individuals, clinical management of FIGO II patients has not been studied well. Hence, we decided to analyse them together with the early-stage OC cases mainly investigated here. Women younger than 18 years of age and women diagnosed with a tumour of low malignant potential or non-epithelial OC were excluded. An overview of sample characteristics is presented in Figure 1 as a flow chart. 

Patient charts, aftercare files, and data stemming from the local tumour registry were used to produce clinical annotations. Patients’ symptoms and location of primary diagnosis (basic/regular care vs. maximum hospital care) and diagnostic procedures (tumour markers and CT/MRT) were documented. Completeness of surgical staging was defined as stated in the German S3 guideline [4], which demands the following procedures: bilateral adnexectomy, hysterectomy, systematic lymphadenectomy, omentectomy, collection of peritoneal fluid, and peritoneal biopsies. Surgical staging was defined as complete when adnexectomy, hysterectomy, systematic lymphadenectomy (except for mucinous disease graded as G1), omentectomy, peritoneal fluid sampling, and peritoneal biopsies (≥1 biopsy sample) were performed. Since information on the mode of surgery (i.e., laparotomic or laparoscopic) had not been collected for this registry, this was not included in the definition of complete surgical staging. For mucinous cancers, appendectomy was considered an obligatory step. The current version of the German guideline states that for early-stage mucinous disease graded as G1, systematic lymphadenectomy is not mandatory as long as no clinical signs of disease are found in the lymph nodes (LNs) [4]. 

The number of positive LNs, location of peritoneal biopsies, histologic subtype, and tumour grade were documented. Although we thoroughly screened tumour databases and patient files, certain information was missing in the final data set. For instance, we could not always exclude that a patient documented as not having had a hysterectomy during cancer surgery might indeed have previously had a hysterectomy in the past. When high- and low-grade cases were compared across histologic subtypes, high grade was defined according to the 2020 definition of the World Health Organisation (WHO): high-grade serous (HGSOC), clear cell (no grading), poorly differentiated (G3) endometroid, and poorly differentiated (G3) mucinous disease. The remaining subtypes and grades were defined as low grades. A total of 9 cases showing serous histology and histologic grade 2 were grouped into the HGSOC subsample. All patients were staged according to the 2014 FIGO staging system for ovarian, fallopian tube, and peritoneal cancer. To increase the validity of the data, FIGO stage was controlled using the documentation of cancer cell spread in completely staged patients. Those with inconclusive FIGO stage were removed from survival analysis and analysis involving FIGO stage (Figure 1). Information on the method of *BRCA* testing (i.e., germ line or somatic) was not available. Furthermore, we documented whether adjuvant chemotherapy had been performed, the chemotherapy protocol, and number of cycles. Finally, follow-up data on relapse-free and overall survival were collected. 

### 2.2. Statistical Methods

Relapse-free survival (RFS) and disease-specific survival (DSS) were defined as the interval between first diagnosis and disease relapse or cancer-related death, respectively. In case of neither cancer-related death nor disease relapse, the data were censored. 

Statistical analysis was performed using SPSS v29 (IBM Corp., Armonk, NY, USA), and data were plotted using SPSS and Microsoft Excel (Microsoft Corp., Redmond, SEA). Sankey-plot was drawn using SankeyMATIC (https://sankeymatic.com/ accessed on 4 March 2023 [8]). The 2-sided *p*-values lower than *p* = 0.05 were considered statistically significant. Pearson’s chi-squared test wase applied to assess whether there was a relationship between two categorical variables. Pair wise comparison of unlinked non-parametric values was performed using Mann–Whitney-U test. To evaluate differences in RFS for statistical significance, log-rank test was employed. The bivariate Kaplan–Meier method was extended to the multivariate Cox regression model to test for association with RFS in the presence of more than one predictor variable.

## 3. Results

### 3.1. Study Sample Characteristics

Initially, data sets were collected from 379 patients that had pT stage pT1-2. During initial data assessment, 22 cases were excluded as they ultimately did not fulfil the inclusion criteria or due to the presence of macroscopically visible peritoneal carcinomatosis outside the pelvis, suggesting an a priori advanced-stage disease (Figure 1). Finally, the data from 357 patients with apparent early-stage OC (i.e., pT1-2 and disease macroscopically limited to the pelvis) were analysed within this retrospective registry (Table 1). For analysing details on surgery, those patients with full medical records on surgical staging (*n* = 317) and apparent pT stage pT1-pT2a (*n* = 292) were selected (Figure 1).

The median patient age was 55.8 years (range 19.1–88.1). The mean follow-up time was 2.0 years. During the follow-up period, 7 patients died of their OC, and the mean DSS was 6.5 years. Due to the rare number of DSS events, survival analysis was only run for RFS. The mean RFS was 5.5 years, and 35 relapses were documented. Most patients were diagnosed with stage pT1a (40.6%; 145/357) or 1c disease (35.3%; 126/357). Regarding histologic subtypes (documented in 353 cases), serous histology was the most common (39.9%; 141/353) followed by endometroid (26.3%; 93/353), mucinous (15.3%; 54/353), and clear cell (13.0%; 46/353) subtypes. Of the serous cases with known information on grade (*n* = 138), 21 patients (15.2%; 21/138) were diagnosed with the low-grade disease. pT stage above pT1 (*p* < 0.001) and high-grade (*p* = 0.036) were prognostic for shortened RFS in the non-stratified cohort. In the multivariate analysis, considering pT, grade/histologic subtype, and age, only the pT stage (*p* = 0.017; HR 5.7) predicted disease recurrence. 

### 3.2. Symptoms, Clinical Setting and Diagnostic Procedures

**Figure 2:**
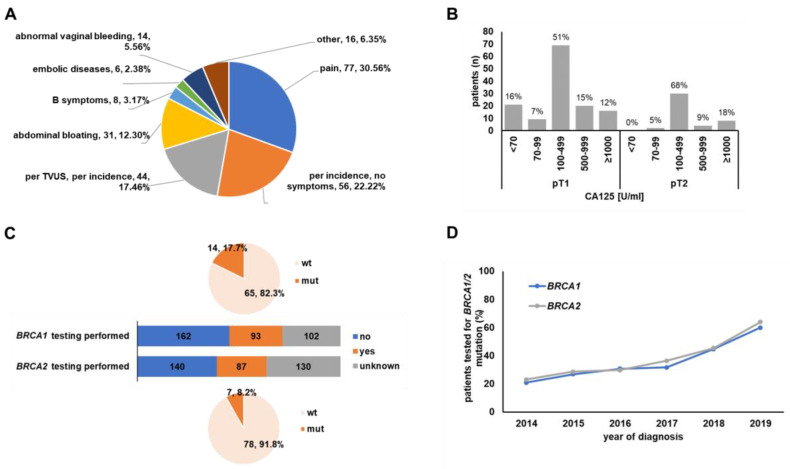
Diagnostics. Prevalence of symptoms is shown in pie chart (**A**). Distribution of CA125 blood levels as split up by pT stage is depicted in bar chart (**B**). Stacked bar charts in C show *BRCA1* and *BRCA2* testing rates. Fraction of patients that underwent *BRCA1* or *BRCA2* testing is marked in orange, while those within the blue fraction were documented to have not undergone *BRCA* testing. Whether *BRCA* testing was performed at all was unknown (not documented) for patients represented by the grey bars. Small pie charts in (**C**) depict *BRCA1*/2 mutation frequency (*BRCA1* upper pie, *BRCA2* lower pie) relative to the cases with known test result of *BRCA1*/2 analysis, respectively. Testing rates for both *BRCA1* and *BRCA2* increased over time (**D**). TVUS = transvaginal ultrasound; wt = wildtype; mut = mutant.

Almost every second patient was diagnosed [42.8% (151/353)] or operated on [41.3% (143/354)] in an external clinic before being admitted to one of the centres running this registry. Seeking a second opinion was documented to be the reason for presentation at our centres in 126 [37.7% (126/334)] cases. Clinical symptoms at the time of initial diagnosis were documented in 252 patients (Figure 2A). Unclear abdominal pain was the symptom most often noted (30.56%; 77/252). Abdominal bloating was another common sign (12.30%; 31/252), while abnormal vaginal bleeding (5.56%; 14/252), B-symptoms (3.17%; 8/252), and embolic disease (2.38%; 6/252) were reported less frequently. Incidental diagnosis or diagnosis via transvaginal ultrasound performed for an unknown reason was documented in 17.46% (44/252) of cases. 

Evaluated serum tumour markers included CA125, HE4, and CEA. HE4 was only determined in 13 cases (10 patients with normal range and 3 with elevated [>2 × ULN] HE4) and CEA in 103 patients (92 patients with normal range and 11 patients with elevated [>2 × ULN] CEA). CA125 was the serum tumour marker most often assessed, with testing performed on 179 patients, and most patients (88.3%) showed clearly elevated (defined as >70 U/mL) CA125 levels (Figure 2B). Though median CA125 levels were not different when pT1 and pT2 stages were compared, CA125 was more commonly found to be significantly elevated (>70 U/mL) in pT2 cases (*p* = 0.005). In addition to tumour marker testing, imaging examinations other than ultrasound were documented for staging in the large majority of patients [79.3% (234/283)].

Whether *BRCA1* or *BRCA2* mutation testing had been performed or not was only known for 255 and 227 cases, respectively. The number of women that indeed had undergone testing was even lower. Only 36.5% (93/255) and 38.3% (87/227) were documented to have been tested for *BRCA1* or *BRCA2* (Figure 2C). Notably, testing rates gradually increased over time (Figure 2D). Test results were recorded in 79 (*BRCA1*) and 85 (*BRCA2*) cases. *BRCA1* mutation was detected in 17.7% (14/79) of patients, and *BRCA2* mutation was detected in 8.2% (7/85) of patients. Neither the diagnostic setting, the *BRCA1*/2 status, nor the CA125 level was of prognostic significance (Supplementary Figure S1).

### 3.3. Surgery

As defined by the inclusion criteria, surgical tumour resection was performed in all patients (*n* = 357). The healthcare level of the hospital where the initial surgery was performed was documented in 335 cases. In 119 of 335 patients (35.5%), the primary surgery was performed in a hospital of basic or regular care level. Whether one- or two-step surgery was performed was known for 349 cases. About one-half of the patients (55.9%; 195/349) received 2-step surgery. In the case of 2-step surgery, the median interval between the first and second operations was 32.5 days. Notably, an interval between the first and second surgery of longer than 60 days was associated with reduced RFS (*p* = 0.018). 

**Figure 3:**
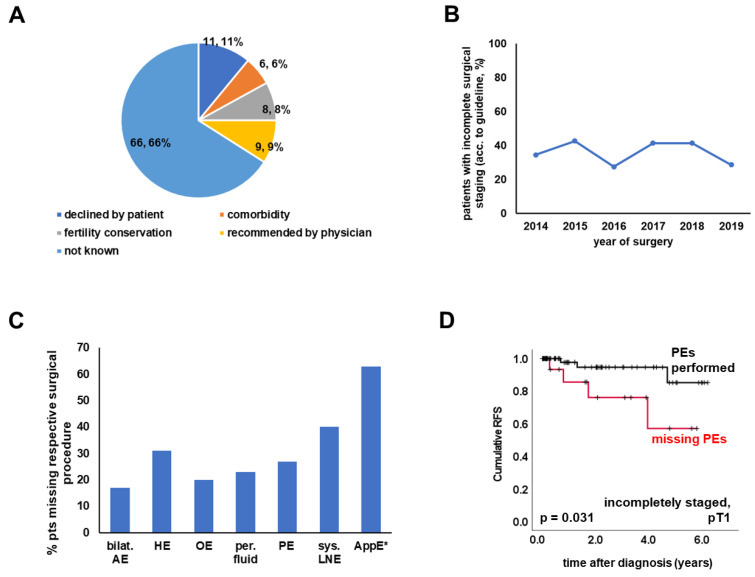
Incompletely Staged Patients. Reasons for incomplete staging are illustrated as a pie chart (**A**). (**B**) shows rate of incomplete surgical staging (i.e., missing at least one surgical procedure recommended, as specified in Materials and Methods) over time. Incompletely staged patients missed at least one surgical step (adnexectomy, hysterectomy, systematic lymphadenectomy (except for mucinous disease graded as G1), omentectomy, peritoneal fluid sampling, peritoneal biopsies (≥1 biopsy sample), or appendectomy (in the case of mucinous subtype)). How often a certain surgical staging step was missed is depicted in (**C**). Missing peritoneal sampling was associated with reduced RFS in incompletely staged cases with pT stage pT1 (**D**, *n* = 87) but failed to be prognostic in the non-stratified cohort (Supplementary Table S1). Bilat. AE = bilateral adnexectomy; HE = hysterectomy; OE = omentectomy; per.fluid = peritoneal fluid sampling; PE = peritoneal biopsies; sys. LNE = systemic lymphadenectomy; AppE = appendectomy; acc = according; * data from mucinous cases only.

Complete medical records on the extent of surgical staging were available for 317 patients (Figure 1). Among these cases, completeness of surgical staging as defined above was analysed in patients staged lower than pT2b (*n* = 292) (Figure 1, Table 2). While 192 (65.8%) were staged according to the interpretation of the current guideline [4] employed in this work (see Method section for details), 100 (34.2%) were missing at least 1 surgical step recommended by the guideline (Figure 1). The reason for breaking with the guideline was documented in only 34 (of 100) cases. Reasons were approximately evenly distributed: declined by the patient (*n* = 11), presence of comorbidities (*n* = 6), fertility conservation (*n* = 8), and recommended by the physician (*n* = 9) (Figure 3A). When comparing patients with complete and incomplete surgical staging, completely staged patients more often had serous histology or were classified as high-grade, while no significant differences regarding pT stage were detected (Table 2). The type of hospital where (primary) surgery was performed did not impact the rate of complete surgical staging. Furthermore, there was no difference regarding the surgical setup (one- vs. two-step), application of chemotherapy, and patient age (Table 2). The rate of incomplete surgical staging over time ranged between 42.6% and 28.6% (Figure 3B).

Within the subgroup of incompletely staged patients (*n* = 100), LN resection was the procedure most often omitted (*n* = 40). Regarding the mucinous subtype, the step most often skipped was appendectomy (63.0%; 17/27; Figure 3C).

The prevalence of missed surgical procedures was not dependent on histology or grade. Hysterectomy was more commonly omitted in cases staged pT1a-c (*p* = 0.035), while peritoneal fluid sampling was more frequently missed in pT2a (*p* < 0.001). Upon comparing incompletely staged patients with one- vs. two-step surgery, those with two-step surgery did less often miss omentectomy (*p* = 0.007) and peritoneal biopsies (*p* = 0.001). Neglecting to take peritoneal samples was prognostic for shortened RFS (*p* = 0.031) in patients that had been assigned pT stage 1 (Figure 3D). In contrast—upon including pT2b—omitting systematic lymphadenectomy was critical (*p* = 0.031) for patients with apparent pT stage pT2a or pT2b. In terms of the non-stratified cohort of incompletely staged patients, the prognostic effect of neglecting to take peritoneal biopsies (*p* = 0.061) or performing systematic lymphadenectomy (*p* = 0.212) did not reach statistical significance (Supplementary Table S1). Neither pT stage nor histology/grade was of prognostic significance in incompletely staged patients. Finally, we analysed whether incomplete surgery impacted patients’ prognosis. Considering all the limitations implicated by the retrospective nature of this analysis and the relative rarity of relapses, we did not observe a significant difference between patients staged completely vs. incompletely. 

**Figure 4:**
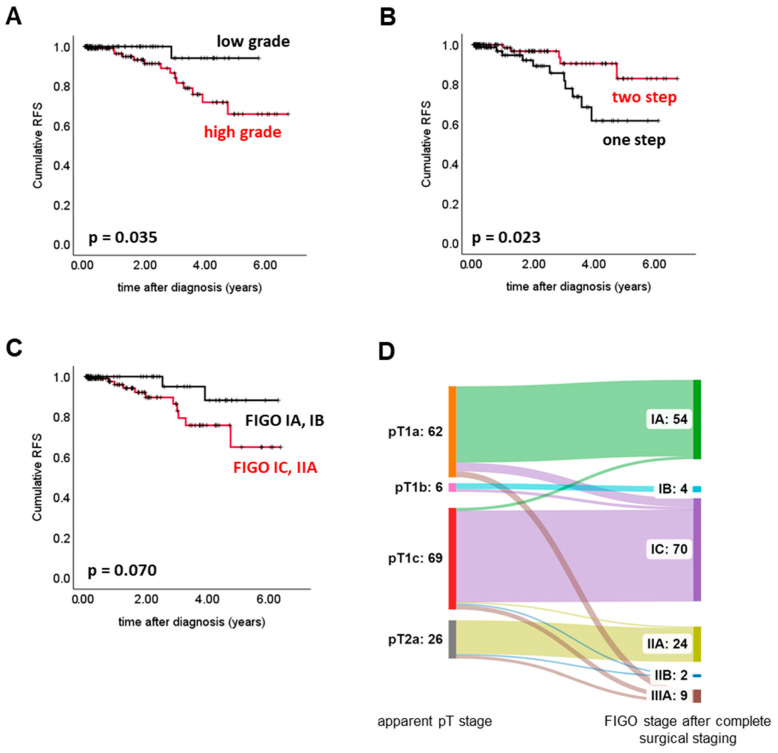
Completely Staged Patients. Survival analyses were performed in completely staged patients with initial pT1a-pT2a stage. High-grade histology (i.e., high-grade serous, clear cell (no grading), poorly differentiated (G3) endometroid, and poorly differentiated (G3) mucinous disease) (**A**) and one-step surgery (**B**) were associated with shortened RFS. Higher FIGO stage (IC and IIA) was of borderline significance only (**C**). pT stage and final FIGO stage (i.e., after complete surgical staging) were linked by employing a Sankey chart (**D**). Complete surgical staging led to upstaging in 20 (12.3%) patients. The most commonly upstaged pT stage was pT1a (*n* = 10), and the FIGO stage most commonly assigned after upstaging was FIGO IIIA (*n* = 9). The number of patients within the respective subgroup with enough data available to perform the calculations shown in (**A**–**D**) was: (**A**) *n* = 187, (**B**) *n* = 191; (**C**) *n* = 162; and (**D**) *n* = 163.

The pattern of cancer spread was analysed in the completely staged cohort. After spreading to ovaries and fallopian tubes (94.3%), peritoneal fluid was the second most affected site (23.5%). LNs were affected in 15 patients (7.9%). LNs and peritoneal fluid were more likely to be affected in the case of serous histology (LN: *p* = 0.002; peritoneal fluid: *p* = 0.004). Potential determinants (Figure 4A,B) for unfavourable RFS were grade/histologic subtype (*p* = 0.035) and one-step surgery (*p* = 0.023). The prognostic value of the FIGO stage was investigated solely in cases for which complete information on the pattern of cancer spread and documented FIGO stage matched. The FIGO stage was found to be of borderline significance (*p* = 0.070, Figure 4C). Intriguingly, one- vs. two-step surgery remained the only parameter that showed prognostic significance (HR = 0.23; *p* = 0.031) in the multivariate analysis. Information on upstaging was available in 163 cases, and complete surgical staging resulted in upstaging in 20 (12.3%) patients. The most commonly upstaged pT stage was pT1a (*n* = 10), and the FIGO stage most commonly assigned after upstaging was FIGO IIIA (*n* = 9) (Figure 4D). Regarding the upstaged cohort, two relapses were documented.

### 3.4. Adjuvant Treatment

Data on adjuvant treatment were available for 296 patients. Those with adjuvant treatment data available and FIGO stage lower than IIB (*n* = 234) were analysed. Most of them received adjuvant chemotherapy (*n* = 170; 72.6%) and had 6 treatment cycles (88.4%; 146 pts with data on treatment cycles) in total. Platinum monotherapy was applied in 96 (53.3%; 180 pts with data on treatment scheme) patients. Patients with high grade (*p* < 0.001), FIGO higher than IB (*p* = 0.001), pT2a (*p* < 0.001), or serous histology (*p* = 0.008) were more likely to receive combination therapy. High-grade cases more often had 6 treatment cycles (*p* = 0.001). Neither the administration of chemotherapy (chemotherapy vs. no chemotherapy), schedule (platinum mono- vs. combination therapy), nor the number of cycles (six cycles vs. less than six cycles) was significantly associated with RFS.

From a quality management point of view, we investigated whether the indication for chemotherapy was given as recommended by the current guidelines. Again, this analysis was run in cases staged in FIGO IA-IIA. Considering FIGO stage and histological grade, three sub-cohorts were defined: ‘adjuvant chemotherapy not recommended’ (FIGO IA, G1); ‘adjuvant chemotherapy recommended’ (G3 or FIGO IC and higher); and ‘adjuvant chemotherapy to be discussed/offered’ (FIGO IA, G2 and FIGO IB G1, G2). Treatment data on 222 cases were available: 6 (2.7%) cases were attributable to “no recommendation”, 185 (83.3%) to “recommendation of chemotherapy”, and 31 (14.0%) to “to be discussed” category. Despite being classified as ‘no recommendation’, one out of the six patients received adjuvant chemotherapy. In contrast, 45 of 185 (24.3%) patients in the ‘recommendation’ group did not receive adjuvant chemotherapy (Supplementary Figure S2). Out of the adjuvant chemotherapy ‘to be discussed’ sub-group, 71.0% (22/31) were actually treated with chemotherapy. Regarding this sub-cohort indication for chemotherapy was not related to histology, grade, or patient age. 

## 4. Discussion

Within this retrospective registry study, we analysed both clinical management and potential prognostic factors for early-stage OC. 

Regarding overall cohort characteristics, the patient sample studied was comparable to those reported in the literature [9]. Most cases were assigned a serous histologic subtype, were graded as high grade and staged as FIGO IA or IC, as described in a large Danish patient cohort [6]. Furthermore, patient age and relapse rates were similar to what has been described by other authors [6,9]. Due to the retrospective nature of this registry, the identification of early-stage OC from the databases was challenging. Therefore, we decided to apply thorough selection criteria to differentiate early- and advanced-stage cases systematically.

As previous studies have described that CA-125 is a poor indicator of early-stage OC [10,11], the diagnostic value of this biomarker in early OC is debatable. Therefore, we analysed the prevalence of elevated CA-125 in our cohort. The data indicated that CA-125 levels were elevated in more than 88% of cases (>70 IU/mL). Whether this discrepancy was due to improved detection levels in laboratory tests in recent years or to assay-specific factors could not be determined. From a clinical perspective and as supported by our data, CA125 might also be useful in apparent early-stage OC (e.g., to monitor treatment response). 

The question of whether the completeness of surgery impacted patient prognosis has yet to be fully addressed. Several studies have aimed to evaluate the impact of surgical staging. Some agree that comprehensive surgical staging is fundamental to adequately diagnosing and treating OC. For example, a 10-year follow-up in the ACTION trial demonstrated that significantly more cancer-specific deaths were observed only among patients staged sub-optimally [12]. Similar observations were reported in a nationwide German registry [5]. In line with this, we confirmed that a lack of both peritoneal biopsies and/or lymphadenectomy increased the risk of disease recurrence. This might be explained by the fact that omitting peritoneal sampling and lymphadenectomy in patients with the apparent macroscopically early-stage disease might lead to misclassification, and as such any microscopic peritoneal or LN metastasis remained undetected. However, this current study did not detect a significant prognostic difference when optimally vs. sub-optimally staged patients were compared. Lacking detection of a difference might also be an issue of too low statistical power. Appendectomy in mucinous subtype cases or hysterectomy, in general, did not have an impact on RFS. Thus, we could speculate that similar to the case in borderline ovarian tumours, hysterectomy might be safely omitted for a certain subgroup of patients (e.g., those who wish to keep their child-bearing potential). Whether this proves to be true needs to be clarified by prospective trials. We observed that established clinicopathological parameters, such as pT-stage and histology/grade, were not prognostic in incompletely staged patients but were in completely staged ones. The observation that such established prognostic markers lose their significance in incompletely staged patients might be taken as an indirect hint that incompleteness of surgical staging might act as a confounder and that complete surgery is relevant for the patient’s prognosis.

It remains to be elucidated whether certain surgical steps might be omitted without losing diagnostic information. As optimal staging is incompatible with a fertility-sparing approach, this increases the perioperative risk and might cause surgery-associated long-term health problems. Consequently, researchers still question whether the extent of surgical staging might be safely reduced, at least for specific patient subgroups. As suggested by the LION trial, systematic resection of LNs not showing any clinical signs of disease did not improve RFS in advanced-stage OC patients [13]. Importantly, these data must not simply translate to early-stage OC, as all the advanced-staged patients in the LION trial received adjuvant chemotherapy, which partly is not the case in early-stage disease. However, independent of the biological and diagnostic significance of microscopic LN metastasis, our increasing knowledge of OC subtypes suggests that there are histologic subtypes with a very low risk of LN metastasis [14]. Regarding the current analysis, the spread to LNs was judged to be a seldom event in low-grade OC. High-grade histology was a risk factor for LN spread, which is in line with the literature [14]. The ongoing LOVE trial (NCT04710797) protocol aims to prospectively dissect the benefit of systematic lymphadenectomy in different histologic subtypes of early-stage OC.

Complete surgery according to the current German S3 guideline (as defined in the Methods section) was performed in 65.8% of cases. This rate is markedly higher than the percentage of comprehensively staged patients reported from a large Danish cohort that comprised more than 1000 patients [6]. An explanation for the low percentage of 3% published by Hengeveld et al. might be that they applied stricter criteria (e.g., the exact localization of biopsies) than we and others did [6]. Le et al. [15] and the nationwide German registry QS Ovar [5] published percentages of completely staged patients in a similar range to ours. Furthermore, the QS Ovar authors also observed lymphadenectomy as the step most often omitted. This might be due to the fact that the indication for lymphadenectomy is incompletely defined by the text of the German S3 guideline and allows some room for interpretation. Moreover, the current study addressed the reasons for incomplete surgery. However, although the original patient files were thoroughly screened, the reason for performing incomplete surgery (e.g., comorbidities, patient’s wish, etc.) was not documented in the majority of cases. Whether this was due to incomplete documentation or the lack of knowledge of the treating physician cannot be determined. In addition, regarding two-step surgery, it might be interesting to analyse which procedures are commonly performed during the first and the second surgery. Unfortunately, our study lacks information on the mode of surgery, i.e., laparotomic, laparoscopic, or robotic approaches. Since minimally invasive surgical techniques are on the increase and seem to bear an increased risk of intraoperative capsule rupture [16], details on surgical methods must be evaluated in upcoming studies/real-world registries.

*BRCA* mutation testing has evolved as standard of care in OC, in Germany up to the age of 80. Therefore, we evaluated the frequency of *BRCA* testing or *BRCA* alterations in our study cohort of early-stage OC. Importantly, although BRCA testing was only performed in a small subpopulation of patients, the frequency of *BRCA1* and *BRCA2* alterations turned out to be similar to what is seen in advanced staged OC [17]. Whether BRCA testing was omitted due to being judged as unnecessary, as PARP inhibitors are not approved for early-stage OC, or the recommendation was overlooked cannot be determined from our data. A total of 3 out of 16 patients diagnosed with a BRCA1 and/or BRCA2 mutation were documented to carry both a mutation in *BRCA1* and *BRCA2* simultaneously. Although cases of double heterozygosity in *BRCA1* and *BRCA2* have been described in the literature, this phenomenon is quite rare and only detected in about 2% of humans [18]. As *BRCA* mutation analysis can be conducted from germline as well as from tumour tissue, rates of double mutation might be slightly higher when combining these two methods. For instance, You et al. report a double mutation/variant of unclear significance in 3 out of 58 ovarian cancer patients [19]. However, our rate of double mutations still seems unexpectedly high, and documentation inaccuracy cannot be fully excluded.

Notably, the frequency of *BRCA* diagnostics increased over time, potentially due to rising awareness, due to the approval of the first PARP inhibitor by the EMA in late 2014 and because it has been reimbursed by most German health insurance companies since 2016. Although our study took place in three large German clinics with extensive oncological experience, *BRCA* testing was still omitted in every third patient in 2019. The absolute number of *BRCA* tests might even be much lower, as testing rates were only estimated for patients for whom information on genetic testing was available (i.e., test performed: yes vs. no). It can be speculated that most patients for whom no information regarding genetic testing was available had not been offered testing. 

Which early-stage OC patients should be recommended to undergo adjuvant chemotherapy is still a matter of debate. The same applies to the question of which chemotherapy protocol should be chosen and the optimal number of treatment cycles. Only a few randomised trials have aimed to resolve these questions; however, patient numbers were small in most investigations, and, therefore, the studies lacked statistical power and/or used chemotherapy protocols no longer applied to OC patients during first-line treatment (e.g., melphalan). Overall, the studies were not able to show a clear benefit of first-line chemotherapy in early-stage OC survival [20,21]. However, these data need to be interpreted with care as there seem to be subgroups of patients that benefit from chemotherapy [22]. The ACTION trial reported that the benefit of adjuvant chemotherapy appeared to be restricted to patients with the suboptimal staging [10]. An extended follow-up analysis of the ICON1 trial concluded that adjuvant chemotherapy should be recommended, especially for those patients with high-risk diseases [23]. However, we did not detect a difference in RFS when the administration of adjuvant chemotherapy was compared. Furthermore, we did not observe a prognostic impact of mono- vs. combination treatment or a number of cycles. Our observations might be biased, as the group of patients who were not treated with adjuvant chemotherapy was relatively small. In addition, the sample size might be too low to detect a statistically significant difference. A recent database analysis run on more than 8500 early-stage ovarian cancer patients demonstrated that delayed start of adjuvant chemotherapy led to reduced overall survival [24]. By showing that delay of the second surgery (which, as a consequence, also postpones the start of chemotherapy) reduces RFS, our data at least indirectly confirm the observation cited above.

By subgrouping the cohort according to their ‘theoretical’ chemotherapy recommendation status as recommended by the S3 German guideline [4], we observed that 24.1% of patients were not treated in accordance with the current guideline. As the reasons for not recommending chemotherapy were not documented, we cannot determine whether chemotherapy was omitted because it was declined by the patient or physician or due to any other reason (e.g., reduced performance status, comorbidities or the age of the patient). Our findings regarding chemotherapy implementation rates indicated that a relevant number of patients were not treated in accordance with the current guidelines. Whether this might be because the role of chemotherapy has not been clearly defined in early-stage OC or for any other reason remains unclear.

The retrospective design of this study and its limitations should be respected when interpreting our results. Importantly, this study has been designed to describe the real-world situation of early OC treatment in the centres running this registry. It should not be used to conclude whether complete surgical staging is necessary or not. However, this current project revealed clinically relevant aspects of early-stage OC management and should be considered a hypothesis-generating study.

Nevertheless, this multicentre database underscores the high need for further prospective registries to increase awareness for adequate clinical management of patients with early-stage OC and to identify better risk factors for relapses. Additionally, the implementation of systematic educational programs is warranted to increase the quality of patient management in the clinical routine.

## 5. Conclusions

This multicentre database underscores the high need for further prospective registries to increase awareness for adequate clinical management of patients with early-stage OC and to identify better risk factors for relapses. Additionally, the implementation of systematic educational programs is warranted to increase the quality of patient management in the clinical routine.

## Figures and Tables

**Table 1:** Patient Characteristics.

	*n* (Data Available)	*n*	% or Range
Patient age (median)		(55.8)	19.1–88.1
pT	357		
1a		145	40.6
1b		14	3.9
1c		126	35.3
2a		42	11.8
2b		30	8.4
Grading	335		
HGSOC		117	34.9
LGSOC		21	6.3
G1		60	17.9
G2		65	19.4
G3		72	21.5
Histology	353		
serous		141	39.9
endometrioid		93	26.3
mucinous		54	15.3
clear cell		46	13.0
mixed		10	2.8
other		9	2.5

Clinicopathological data of patients diagnosed with apparent early-stage epithelial ovarian cancer (second top box in Figure 1, *n* = 357) are shown. HGSOC = high-grade serous ovarian cancer; LGSOC = low-grade serous ovarian cancer.

**Table 2:** Clinicopathological Characteristics of Patients with Complete vs. Incomplete Surgical Staging (stages pT1-pT2a only).

		Surgical Staging		
	Data Available (*n*)	Complete	Incomplete	*p*
pT				
pT1	292	165	91	ns
pT2		27	9	
Histology				
other	291	110	71	0.016
serous		82	28	
Grade				
low	281	68	48	0.013
high		120	45	
(Primary) Surgery				
regular care hospital	291	130	58	ns
maximal care hospital		62	41	
Surgical Setup				
one-step	291	85	43	ns
two-step		107	56	
Received CHT				
no	243	135	54	ns
yes		36	18	
Patient Age				
≤56 years	288	104	46	ns
>56 years		87	51	

Completeness of surgical staging (complete vs. incomplete) was tested for independence from pT, histology, grade, location of (primary) surgery, surgical setup, application of chemotherapy (CHT), and patient age. *p*-values derived from Chi2 tests are displayed. ns = not significant

## Data Availability

All relevant data are presented in the manuscript.

## Supplementary Materials

The following supporting information can be downloaded at: https://www.mdpi.com/article/10.3390/cancers15072038/s1, Figure S1: Prognostic value of CA125, BRCA mutation and clinic where the (first) surgery was performed. Neither CA125 (A), *BRCA* mutation (B) nor location of (first) surgery were prognostic for RFS; Figure S2: Implementation of adjuvant chemotherapy is shown as Sankey diagram. Considering FIGO stage and histological grade, three theoretical sub-cohorts were defined: ‘adjuvant chemotherapy not recommended’ (FIGO IA, G1); ‘adjuvant chemotherapy recommended’ (G3 or FIGO IC and higher); and ‘adjuvant chemotherapy to be discussed’ (FIGO IA, G2, and FIGO IB G1, G2) (left column). Theoretical sub-cohorts (left column) and clinical practice (right column) are connected by flows of a Sankey chart; Table S1: Comparison of PFS in incompletely staged patients that had not vs. had undergone peritoneal biopsies/systemic lymphadenectomy.
