# Supplementary material for: Current Treatment Practices and Prognostic Factors in Early-Stage Ovarian Cancer—An Analysis of the NOGGO/JAGO
Source: Cancers (Basel). 2023 Mar 29;15(7):2038. doi: 10.3390/cancers15072038 (PMC10093615; doi:10.3390/cancers15072038)
Supplement: Supplementary file 1 [file cancers-15-02038-s001.zip › cancers-2232740-supplementary.pdf]

Current Treatment Practices and Prognostic Factors in Early-Stage Ovarian Cancer—An Analysis of the NOGGO/JAGO

Sabine Heublein [1,2,3,*], Joanna Baum [3,4], Anna Jaeger [3,5], Donata Grimm-Glang [3,6], Julia Olthoff [3,7], Elena Ioana Braicu [4], Osama Azzam Nieto [1,3], Kathrin Hassdenteufel [1], Barbara Schmalfeldt [5], Lars Hanker [6], Markus Wallwiener [1], Andreas Schneeweiss [2], Jalid Sehouli [4] and Klaus Pietzner [3,4]

1 Department of Gynecology and Obstetrics, University of Heidelberg, 69120 Heidelberg, Germany
2 National Center for Tumor Diseases, University Hospital and German Cancer Research Center Heidelberg, 69120 Heidelberg, Germany
3 Young Academy of Gynecologic Oncology (JAGO), 13359 Berlin, Germany
4 Department of Gynecology, Center for Oncological Surgery, Campus Virchow Klinikum, Charite-Universitätsmedizin Berlin, Corporate Member of Freie Universität Berlin, Humboldt-Universität zu Berlin, Berlin Institute of Health, 13353 Berlin, Germany
5 Department of Gynecology and Gynecologic Oncology, University Medical Center Hamburg-Eppendorf, 20246 Hamburg, Germany
6 Department of Obstetrics and Gynecology, University Medical Center Luebeck, 23562 Luebeck, Germany
7 Department of Obstetrics and Gynecology, University Hospital Brandenburg an der Havel, 14770 Brandenburg an der Havel, Germany
* Correspondence: sabine.heublein@med.uni-heidelberg.de

Supplementary Data

Supplementary Table S1. Comparison of PFS in incompletely staged patients that had not vs. had undergone peritoneal biopsies / systemic lymphadectomy.

	pT1-pT2a		pT1-pT2b		pT1a-c		pT2a, b	
	HR	p	HR	p	HR	p	HR	p
peritoneal biopsies (no vs. yes)	0.26	0.061	0.35	0.085	**0.22**	**0.031**	1.81	0.633
systematic LNE (no vs. yes)	1.17	0.85	0.46	0.212	1.24	0.801	**0.01**	**0.031**

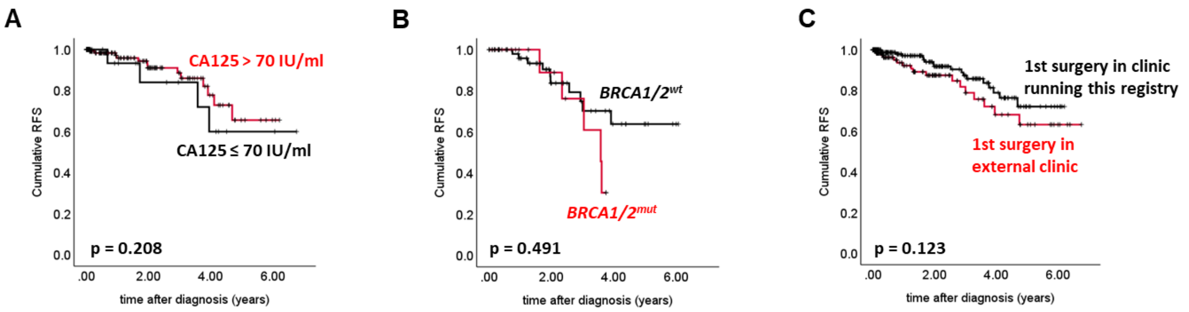

Supplementary Figure S1. Prognostic value of CA125, BRCA mutation and clinic where the (first) surgery was performed. Neither CA125 (A), *BRCA* mutation (B) nor location of (first) surgery (C) were prognostic for RFS.

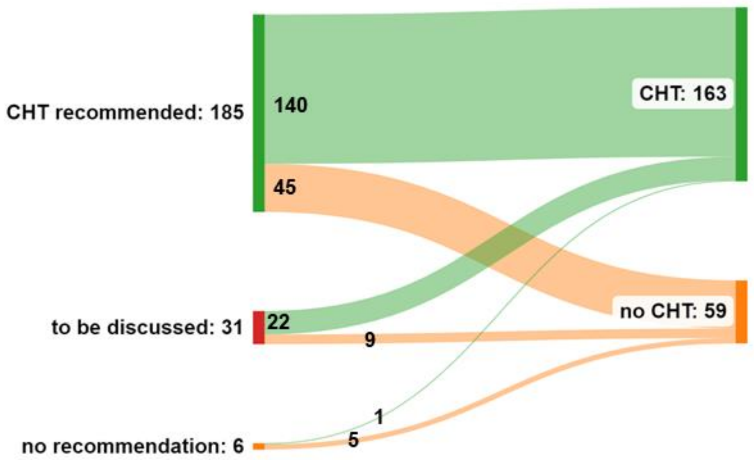

Supplementary Figure S2. Implementation of adjuvant chemotherapy is shown as Sankey diagram. Considering FIGO stage and histological grade, three theoretical sub-cohorts were defined: 'adjuvant chemotherapy not recommended' (FIGO IA, G1); 'adjuvant chemotherapy recommended' (G3 or FIGO IC and higher); and 'adjuvant chemotherapy to be discussed' (FIGO IA, G2, and FIGO IB G1, G2) (left column). Theoretical sub-cohorts (left column) and clinical practice (right column) are connected by flows of a Sankey chart.
